# Antioxidant Treatment with N-acetyl Cysteine Prevents the Development of Cognitive and Social Behavioral Deficits that Result from Perinatal Ketamine Treatment

**DOI:** 10.3389/fnbeh.2017.00106

**Published:** 2017-06-06

**Authors:** Aarron Phensy, Hasmik E. Duzdabanian, Samantha Brewer, Anurag Panjabi, Christopher Driskill, Annuska Berz, George Peng, Sven Kroener

**Affiliations:** School of Behavioral and Brain Sciences, The University of Texas at DallasRichardson, TX, United States

**Keywords:** ketamine, oxidative stress, cognitive flexibility, novel object recognition, social interaction, spontaneous alternation, amphetamine-induced hyperlocomotion, prepulse inhibition

## Abstract

Alterations of the normal redox state can be found in all stages of schizophrenia, suggesting a key role for oxidative stress in the etiology and maintenance of the disease. Pharmacological blockade of N-methyl-D-aspartic acid (NMDA) receptors can disrupt natural antioxidant defense systems and induce schizophrenia-like behaviors in animals and healthy human subjects. Perinatal administration of the NMDA receptor (NMDAR) antagonist ketamine produces persistent behavioral deficits in adult mice which mimic a range of positive, negative, and cognitive symptoms that characterize schizophrenia. Here we tested whether antioxidant treatment with the glutathione (GSH) precursor N-acetyl-cysteine (NAC) can prevent the development of these behavioral deficits. On postnatal days (PND) 7, 9 and 11, we treated mice with subanesthetic doses (30 mg/kg) of ketamine or saline. Two groups (either ketamine or saline treated) also received NAC throughout development. In adult animals (PND 70–120) we then assessed behavioral alterations in a battery of cognitive and psychomotor tasks. Ketamine-treated animals showed deficits in a task of cognitive flexibility, abnormal patterns of spontaneous alternation, deficits in novel-object recognition, as well as social interaction. Developmental ketamine treatment also induced behavioral stereotypy in response to an acute amphetamine challenge, and it impaired sensorimotor gating, measured as reduced prepulse inhibition (PPI) of the startle response. All of these behavioral abnormalities were either prevented or strongly ameliorated by NAC co-treatment. These results suggest that oxidative stress is a major factor for the development of the ketamine-induced behavioral dysfunctions, and that restoring oxidative balance during the prodromal stage of schizophrenia might be able to ameliorate the development of several major symptoms of the disease.

## Introduction

Schizophrenia is a neurodevelopmental disorder in which genetic risk factors and early life stressors converge. Early postnatal blockade of N-methyl-D-aspartic acid (NMDA) receptors provides a widely used rodent model for studying the neurodevelopmental consequences of NMDA receptor (NMDAR) dysfunction, which are at the center of glutamate hypotheses of schizophrenia (Javitt and Zukin, 1991; du Bois and Huang, 2007; Castañé et al., 2015). These hypotheses developed out of the observation that NMDAR antagonists induce core symptoms of the illness, including cognitive deficits, both in healthy humans (Javitt and Zukin, 1991; Krystal et al., 2002) and in animals (Amitai et al., 2007; Coleman et al., 2009; Chatterjee et al., 2011), and that they exacerbate symptoms in schizophrenic patients (Lahti et al., 1995; Malhotra et al., 1997). Cognitive dysfunction and negative symptoms of schizophrenia (Kahn and Keefe, 2013) are a major determinant of functional outcome in the disease (Green, 1996), but are poorly responsive to available medications (Keefe and Harvey, 2012). Brains of schizophrenic patients show signs of redox dysregulation/oxidative stress, and reduced levels of antioxidants, including glutathione (GSH), which provide endogenous protection against reactive oxygen species (ROS) and reactive nitrogen species (RNS; Gawryluk et al., 2011; Do et al., 2015). Oxidative and nitrosative stress are the result of an imbalance between the overproduction of ROS and RNS on one side, and a deficiency of enzymatic and non-enzymatic antioxidants such as GSH on the other. The brain is particularly vulnerable to oxidative stress due to its high content of polyunsaturated fatty acids, high oxygen consumption, and low levels of antioxidant enzymes (Bitanihirwe and Woo, 2011; Möller et al., 2015). Oxidative stress during development may lead to misconnectivity in specific brain regions and dysfunctional integration of sensory information relevant for cognitive processes (Bitanihirwe and Woo, 2011). Both peripheral tissue and neurons in the CNS of schizophrenic patients show oxidative stress and abnormal levels of antioxidants, including GSH (Gysin et al., 2007; Gawryluk et al., 2011; Do et al., 2015). Deregulation of ROS is correlated with negative symptoms and cognitive deficits in schizophrenia. For example, deficits in attention, as well as both immediate and delayed memory are inversely correlated with plasma total antioxidant status (Zhang et al., 2012, 2013). Similarly, there is a negative correlation between brain GSH levels and the severity of negative symptoms, which include blunted affect, lack of volition, and social withdrawal (Berk et al., 2008; Matsuzawa and Hashimoto, 2011). Such studies have renewed interest into antioxidants as a potential treatment (Do et al., 2015). NMDAR antagonists can trigger rapid increases in ROS *in vivo* (Zuo et al., 2007; Powell et al., 2012a), and thus NMDAR dysfunction is likely to contribute to the oxidative stress seen in schizophrenia (Mahadik and Mukherjee, 1996; Prabakaran et al., 2004). Cysteine is the rate-limiting substrate for the synthesis of GSH, and thus supplementation of N-acetyl-cysteine (NAC) promotes the resynthesis of GSH and the neutralization of free radicals. GSH has antioxidant properties by itself, and in addition, it facilitates the activity of enzymatic antioxidant systems, including glutathione peroxidase (GPx) and GSH reductase. Reduced levels of GSH or GPx are correlated with impaired synaptic plasticity and cognition (Almaguer-Melian et al., 2000; Massaad and Klann, 2011). In animal models of schizophrenia, NAC has already been successfully used to prevent changes in sensory gating functions (Lutgen et al., 2013; Cabungcal et al., 2014), as well as changes in parvalbumin-expressing interneurons, which are believed to be at the hub of many network changes in the brains of schizophrenic patients (Lewis et al., 2012). However, the effects of NAC on cognitive performance and other dimensions that may be affected in schizophrenia have not been extensively studied. The Measurement and Treatment Research to Improve Cognition in Schizophrenia (MATRICS) initiative established by the National Institute of Mental Health (NIMH) identified seven cognitive domains that are impaired in schizophrenia: attention/vigilance, working memory, reasoning and problem solving, processing speed, visual learning and memory, verbal learning and memory and social cognition (Marder, 2006). A number of tasks have been validated in rodents to measure equivalent cognitive dimensions in animal models of the disease (reviewed in Young et al., 2009, 2012; Amann et al., 2010). Transient NMDAR blockade during development with PCP, MK-801, or ketamine induces persistent impairments in cognitive tasks, including novel object recognition (as a rodent measure of visual episodic memory), delayed spontaneous alternation (as a measure of spatial short-term memory or working memory), sensorimotor gating of the startle effect (as a measure of preattentional information processing), attentional set-shifting or cross-maze rule-shifting tasks (as measures of reasoning and problem solving), and social interaction (as a measure of social withdrawal among the negative symptoms of schizophrenia; Stefani and Moghaddam, 2005; Terranova et al., 2005; Harich et al., 2007; Wedzony et al., 2008; Young et al., 2009; Jeevakumar et al., 2015; Grayson et al., 2016). Here, we used variants of these tasks to test whether antioxidant treatment with NAC can prevent the development of behavioral deficits that follow perinatal NMDAR blockade with ketamine (Jeevakumar et al., 2015). Mice were treated with saline or ketamine (30 mg/kg) on postnatal days (PND) 7, 9 and 11, followed by either saline- or NAC-treatment throughout development. We then tested adult animals from the 4 treatment groups for differences in performance on novel object recognition, spontaneous alternation, social interaction, a rule-shifting task, and prepulse inhibition (PPI). In addition, we assessed ketamine- and NAC-induced changes in amphetamine-induced locomotion in an open field as a model for a hyperdopaminergic state that can contribute to positive symptoms of schizophrenia.

## Materials and Methods

### Animals and Drug Treatments

**Figure 1:**
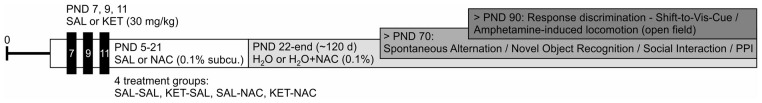
Experimental time line. Starting on postnatal day (PND) 5, animals received subcutaneous injections of either N-acetyl-cysteine (NAC) or equivalent amounts of saline (SAL). On PND 7, 9 and 11 animals additionally received injections of either ketamine (KET; 30 mg/kg) or saline, resulting in four treatment groups (SAL-SAL, KET-SAL, SAL-NAC, KET-NAC). Daily saline or NAC injections continued until animals were weaned on PND 21; from there on animals in the NAC groups received NAC (0.1% by volume) in their drinking water until the end of the experiments around PND 120. Animals in the other groups drank unadulterated water. Animals were tested in four different cohorts (see text for details). Testing for Spontaneous Alternation, Novel Object Recognition, Social Interaction and Prepulse Inhibition (PPI) began after PND 70. Food restriction for Response Discrimination and Shift-to-Visual-Cue Discrimination started after PND 90. Similarly, amphetamine-induced locomotor activity was also tested after PND 90.

Male mice of the G42 line (CB6-Tg(Gad1-EGFP)G42Zjh/J; Jackson Laboratories, Bar Harbor, ME, USA RRID:IMSR_JAX:007677) were used for all experiments. Littermates were assigned to one of four groups: Saline-control (SAL-SAL), ketamine followed by saline (KET-SAL), ketamine followed by NAC (KET-NAC), or saline followed by NAC (SAL-NAC). Starting on PND 5, all animals received subcutaneous injections of either saline or NAC (0.1% in saline; 10 μl/g bodyweight) until they were weaned at PND 21. In addition, on PND 7, 9 and 11 all animals received subcutaneous injections of either saline, or a subanesthetic dose of ketamine (30 mg/kg; Ketathesia HCl, Henry Schein) to induce NMDAR dysfunction. After weaning, animals in the NAC groups continued to receive NAC (0.1%) in their drinking water until the end of the experiments. All experiments were performed using adult animals (PND 70–120). Figure 1 shows a graphical summary of the experimental time course. We used different cohorts of animals for the different behavioral tasks: one cohort of animals was used for the spontaneous alternation task, novel object recognition, and the social interaction task. For these experiments the number of animals in each treatment group ranged between 8–10 mice. Three other cohorts of animals were tested separately either on the Response Discrimination and Shift-to-Visual-Cue Discrimination (group sizes of 8–10 animals), for their locomotor response following an amphetamine challenge in an open field (group sizes of 13–17 animals), or for PPI (group sizes of 11–14), respectively. Prior to all behavioral tasks animals were handled for 2 weeks in their home cages for 5 min each day. In addition, 3 days before the experiments animals were also handled in the room where the experiments were performed. On the day of testing, animals were transferred to the behavioral room at least 30 min before testing began. Experimenters were blind to the treatment of experimental animals throughout testing and analysis. All procedures were approved by the Institutional Animal Care and Use Committee of The University of Texas at Dallas.

### Response Discrimination and Shift-to-Visual-Cue Discrimination

**Figure 2:**
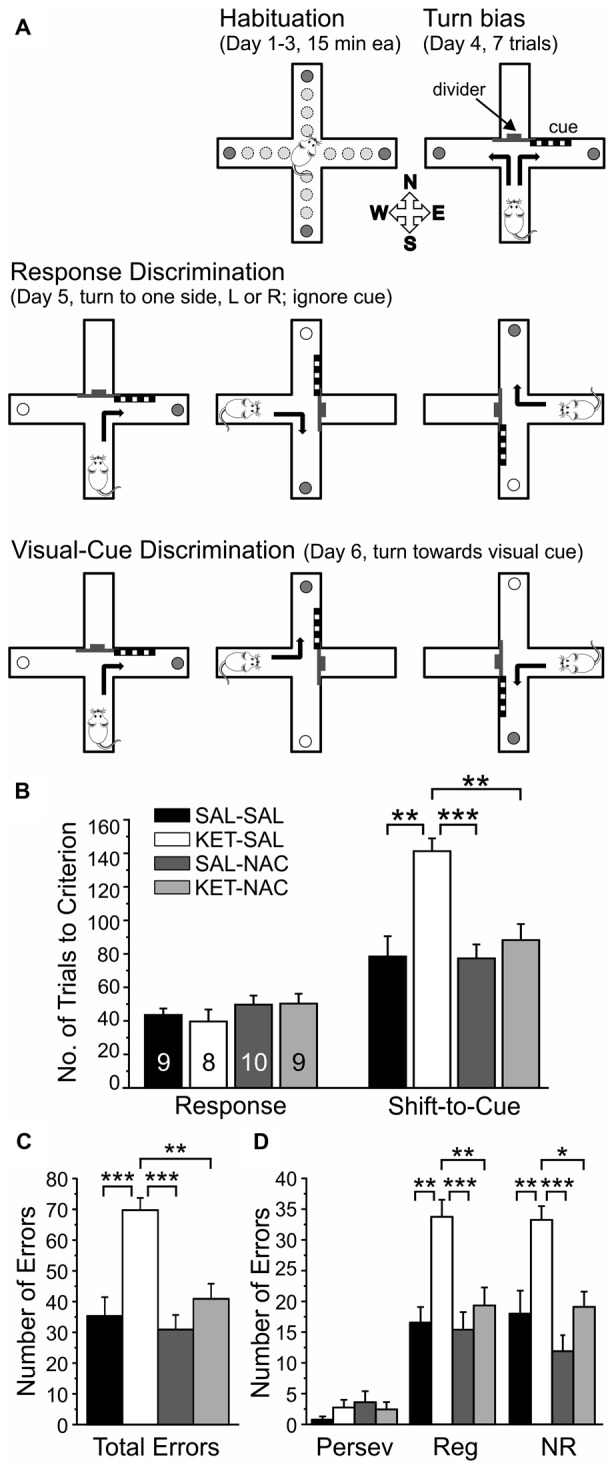
NAC treatment prevents deficits in cognitive flexibility that result from perinatal ketamine treatment. **(A)** Illustration of the experimental setup and design. Animals were habituated to the maze over 3 days. On the fourth day, animals were tested for an innate preference to turn left or right in the T-maze (Turn bias). On the next day, animals were then trained against their turn bias to perform an egocentric response discrimination in order to retrieve a food reward in one of the two arms of the T-maze. Training took place in the presence of a visual cue that was placed pseudorandomly in a balanced manner into one of the two arms. On the final day, the animals were required to shift their strategy, so that they now always had to turn into the arm that contained the visual cue to obtain the reward. **(B)** Animals in all treatment groups acquired the strategy for the Response Discrimination (Response) at the same rate. In contrast, ketamine-treated animals required significantly more trials to criterion in the Shift-to-Visual-Cue Discrimination (Shift-to-Cue). Both SAL-NAC and KET-NAC animals performed comparable to controls. **(C)** KET-SAL animals committed significantly more errors vs. controls while shifting to the visual cue-based strategy. Mice receiving NAC-treatment did not exhibit this increase in errors, performing comparable to controls. **(D)** Error types were further defined as perseverative (Persev), regressive (Reg) and never reinforced (NR). Ketamine treatment induced significant increases in both regressive- and never-reinforced errors, suggesting that the treatment impairs the ability to learn and maintain a second strategy, which can be prevented by NAC co-treatment. Significance is indicated as * for *P* < 0.05, ** for *P* < 0.01, and *** for *P* < 0.001, following Bonferroni correction.

Procedures followed those previously described (Jeevakumar et al., 2015), which are modified from Floresco et al. (2006). Mice were gradually food restricted to 85% of their free-feeding weight over 2 weeks. The task was performed over the course of 6 days. In brief, mice were habituated to a plus maze over 3 days and learned to explore all four arms to retrieve food rewards. On day 4, the maze was converted to a T-maze and the propensity of mice to turn left or right (turn bias) was assessed. On day 5, the mice were trained on a response discrimination that required them to always turn to one side (against their turn bias). Finally, on day 6 mice had to shift strategies and turn towards a previously ignored visual cue to obtain the reward. Figure 2A illustrates the experimental setup on the different days of training.

### Apparatus

Testing took place in white wooden plus maze (each arm is 10 × 34 × 15 cm, with a 10 × 10 cm center area) under low ambient illumination. The arms were labeled East, West, South and North for reference. On days 4–6, the maze was converted into a T-maze by blocking off one of the arms with a divider, and additionally a visual cue (vertical black and white stripes on a 13 × 10 cm plastic sheet) was placed alternately near the entrance of one of the two choice arms in a pseudorandom manner (see below). During all days, reward pellets (Cheerio bits) were placed around the outside of the maze in order to prevent animals from using olfactory cues to infer the location of the reward.

### Habituation (Day 1–3)

On the first day of habituation, four reward pellets (1/8th Cheerios bits) were placed in each of the arms of the plus maze. Animals were placed into the center of the maze and were allowed to freely explore the maze for 15 min. If a mouse consumed all 16 pellets before the end of the 15 min habituation period, it was briefly placed in a holding cage while the maze was rebaited, and then the mouse was placed back into the maze until the end of the 15 min period. On the second day of habituation, arms were baited with two pellets each, and on the third day of habituation only one food pellet was placed at the end of each arm. To reach habituation criterion, animals were required to consume all four food pellets at least four times within the 15 min period. All animals in this study reached this criterion on the third habituation day.

### Turn Bias (Day 4)

On the following day, the animals’ turn bias was determined. Therefore, the plus maze was converted into a T-maze by blocking off one of the arms. Mice were placed in the stem arm and allowed to turn left or right to obtain a food pellet. After the mouse consumed the reward, it was returned to the stem arm and allowed to make another choice. If the mouse chose the same arm as on the initial choice, it was returned to the stem arm until it chose the other arm and consumed the food pellet. The direction of the initial turn chosen four or more times over seven trials was considered the turn bias.

### Response Discrimination (Day 5)

On the next day, mice were trained on a response discrimination task that required them to always turn into only one arm (left or right, chosen opposite to the direction of their turn bias) to obtain the food reward. The location of the stem arm was pseudorandomly rotated among three arms (East, West and South) to prevent mice from using an allocentric spatial strategy to locate the food. A visual cue was placed close to the entrance of one of the choice arms. Placement of this cue into the right or left arm varied pseudorandomly to balance the frequency of occurrences in each arm across blocks of 12 consecutive trials. Similarly, the order of the stem arms alternated pseudorandomly in a balanced fashion across blocks of 12 trials. Training continued until the mouse made nine correct choices over 10 consecutive trials. When animals achieved this acquisition criterion, a probe trial was administered. In the probe trial the previously unused fourth arm (North) was used as a stem arm. If the mice performed the probe trial correctly, Response Discrimination training was completed. If an incorrect turn occurred, response training continued until the mouse made another five consecutive correct choices, and then another probe trial was administered.

### Shift-to-Visual-Cue Discrimination (Day 6)

On the final day, mice were trained to shift their strategy to now follow the visual cue in order to obtain food rewards. The location of the visual cue and the position of the start arm were again varied pseudorandomly so that their frequency was balanced across blocks of 12 consecutive trials. The training and response criteria for the Shift-to-Visual-Cue Discrimination were identical to those during Response Discrimination.

### Performance and Error Analysis

For each of the two test days, we analyzed the total number of trials to criterion and the number of probe trials required to reach criterion. For the Shift-to-Visual-Cue Discrimination, errors were scored as entries into arms that did not contain the visual cue, and they were further broken down into three subcategories to determine whether the animals’ treatment altered the ability to either shift from the previously learned strategy (*perseverative errors*), or to maintain the new strategy after perseveration had ceased (*regressive errors*, or *never-reinforced errors*). In order to detect shifts in the strategies animals used, trials were separated into consecutive blocks of four trials each. A *perseverative error* occurred when a mouse made the same egocentric response as required during the Response Discrimination, but which was opposite to the direction of the arm containing the visual cue. Six of every 12 consecutive trials required the mouse to respond in this manner. A perseverative error was scored when the mouse entered the incorrect arm on three or more trials per block of 4 trials. Once the mouse made less than three perseverative errors in a block, all subsequent errors of the same type were now scored as *regressive errors* (because at this point the mouse was following an alternative strategy at least half of the time). So-called *never-reinforced errors* were scored when a mouse entered the incorrect arm on trials where the visual cue was placed on the same side that the mouse had been trained to enter on the previous day.

### Delayed Spontaneous Alternation

Mice were placed into the stem arm of a T-maze and a central partition wedge was placed in between the horizontal goal arms. Mice were left to choose between the left and the right open goal arm. Once a mouse had entered an arm it was confined to this arm for 30 s by blocking off the entrance of the arm. After 30 s, the barrier was removed and the animal was placed back into the stem arm and allowed to once more choose between the left and right arm. Each animal completed a total of six trials with a 20 min interval between trials. The maze was cleaned with 70% ethanol after each trial. Each trial was timed and the direction of the turns was recorded to calculate a percentage of spontaneous alternations between left and right arm entries.

### Novel Object Recognition

Mice were first habituated for 2 days to an empty open chamber (39 × 19 × 30.5 cm) in 10 min sessions. On the third day, the 10 min habituation session was followed by a training trial during which mice were placed back into the chamber and were allowed to investigate two wooden objects for 3 min. Animals were then placed into their home cage for 2 min before they were placed back into the chamber which now contained one familiar object from the training trial and one novel wooden object. Animals again were allowed to investigate both objects for 2 min. The objects were cleaned with 20% ethanol and the chamber was cleaned with 70% ethanol between all trials, and configurations of the objects during each trial were changed for each animal in all treatment groups. Both the training trial and novel trial were recorded by an overhead camera and the amount of time the mice spent investigating the objects during both the training trial and the novel object trial were analyzed. In order to assess whether animals recognized the novel object as such we calculated a “recognition index” which is the percentage of time spent investigating the novel object over the total investigation time for both objects.

### Social Interaction

Experimental mice and two stimulus mice were housed individually for 3 days prior to the task. On test day, the experimental mice were placed in a new cage for 1 h prior to experimentation. A stimulus mouse was placed in a custom cylindrical cage (height 20 cm, steel bars separated by 1 cm, acrylic base and lid) and introduced into the cage of the experimental mouse for four consecutive trials. Trials lasted 1 min each and were separated by 10 min intervals during which the stimulus mouse was returned to its home cage. For the fifth trial, a second stimulus mouse was placed in the custom cage and introduced to the experimental animal’s cage for 1 min. The cylindrical cages were cleaned with 20% ethanol after each exposure. All trials were video recorded and interaction times (defined as sniffing and investigation at close proximity) were scored.

### Amphetamine-Induced Locomotor Activity

Mice were placed inside a 60 × 60 cm open field and their behavior was video-recorded over a 30 min baseline period using an overhead camera and two side mounted cameras. After the baseline period, mice were given an i.p. injection of amphetamine (4 mg/kg d-amphetamine hemisulfate; Sigma, St. Louis, MO, USA) in saline. They were placed in their home cage for 10 min before they were returned to the open field for an additional 60 min. The total distance traveled was measured using a custom-written MATLAB program (Rodent tracker, Vulintus, Plano, TX, USA). In order to assess changes in stereotypic behavior, three 1-min clips of baseline activity and 20 1-min clips of post-injection activity (selected at 3 min intervals) were rated by two investigators using a 1–9 scale: 1—asleep: lying down, eyes closed; 2—inactive: lying down, eyes open; 3—in-place activities: normal grooming or chewing cage litter; 4—normal: alert, active moving about cage, sniffing, rearing; 5—hyperactive: running, movement characterized by rapid changes in position (jerky), 6—slow patterned: repetitive exploration of the cage at normal level of activity; 7—Fast patterned: repetitive exploration of the cage with hyperactivity; 8—restricted: remaining in same place in cage with fast repetitive head and/or foreleg movement (includes licking, chewing and gnawing stereotypies); and 9—dyskinetic-reactive: backing up, jumping, seizures abnormally maintained postures, dyskinetic movements (Ellinwood and Balster, 1974).

### Prepulse Inhibition of the Acoustic Startle Response (PPI)

Mice were tested in an acrylic cage (9 × 5.5 × 4.5 cm) with a wire mesh bottom contained within an insulated startle box (67 × 67 × 67 cm). The cage was mounted on a startle platform (Lafayette Instrument Co., Lafayette, IN, USA) that uses a piezoelectric transducer to generate a continuous record of activity measured in volts. Startle tones were generated using a real-time processor (RP2.1, Tucker-Davis Technologies, Alachua, FL, USA) and presented by an overhead speaker (Optimus Bullet Horn Tweeter) positioned above the cage offset 20 cm from the center of the cage. Mice were habituated to the startle box twice a day for 2 days in 5 min sessions with 65 dB white noise playing in the background. On day 3, an input-output curve of the startle response was recorded. Mice were placed into the startle box and given a 5-min acclimation period during which only the 65 dB background noise was played. Following the acclimation period, the animals were presented with a startle tone (20 ms white noise) every 20 s, starting at 75 dB and increasing in loudness by 5 dB until a maximum of 115 dB was reached, totaling 10 startle presentations. The peak-to-peak voltage generated by the startle platform within 500 ms following the startle tone was recorded for each startle presentation. These points were then normalized for each animal using the following equation: $x_{\text{i}}\;=\;\frac{x_{\text{i}}-x_{\min}}{x_{\max}-x_{\min}}.$ On day 4, mice were placed into the startle box and given a 5-min acclimation period during which only the 65 dB background noise was played. This was immediately followed by 20 presentations of the startle tone (20 ms, 115 dB) by itself in order to establish the baseline startle for the animal. Animals were then presented with a prepulse tone (4 ms white noise; 75 dB or 85 dB) followed by a startle tone with a 30 ms interstimulus interval. Trials were pseudorandomly ordered by prepulse intensity, and each prepulse intensity was presented 10 times each with a 20 s intertrial interval. Peak-to-peak voltage startle responses were recorded for each presentation. To analyze PPI, prepulse trials were sorted by prepulse intensity and then the startle response was averaged across all 10 trials for each intensity. PPI was calculated by dividing the prepulse startle over the baseline startle for each animal and subtracting this from 100%. Animals with low baseline startle, or animals that were moving during most of the test period were excluded. A total of 11 (3 SAL-SAL; 3 KET-SAL; 2 SAL-NAC; 3 KET-NAC) out of 51 animals were excluded based on these criteria.

### Statistical Analysis

Differences between groups were compared using one-way ANOVAs or two-way mixed ANOVAs as indicated. *Post hoc* analyses using Bonferroni correction were used to determine specific group differences. For all measures the data is presented as mean ± standard error of the mean (SEM). An alpha level of *p* < 0.05 was considered significant.

## Results

### Response Discrimination and Shift-to-Visual-Cue Discrimination

Deficits in cognitive flexibility mediated by the PFC represent a significant disability associated with schizophrenia and several other neurological and neuropsychiatric disorders (Pantelis et al., 1999; Nieuwenstein et al., 2001). In order to test the effects of NAC on deficits in cognitive flexibility induced by perinatal ketamine treatment, we studied differences in the acquisition of a Response Discrimination, followed by a Shift-to-Visual Cue-Discrimination (Floresco et al., 2006; Jeevakumar et al., 2015). This task required the animals to shift between two strategies and in the second discrimination to focus on a previously ignored stimulus in order to receive a reward (Figure 2A). A one-way ANOVA revealed that animals in all treatment groups were able to acquire the Response Discrimination strategy at the same rate (*F*_(3,32)_ = 0.811, *p* = 0.497; Figure 2B). In contrast, drug treatment significantly affected the number of trials required to shift to the visual cue strategy (*F*_(3,32)_ = 9.267, *p* < 0.001; Figure 2B). *Post hoc* comparisons using Bonferroni correction showed that KET-SAL treated animals took significantly more trials compared with controls, while both NAC treated groups performed comparable to controls. A separate ANOVA also revealed an effect of treatment on total number of errors committed during the Shift-to-Visual-Cue Discrimination (*F*_(3,32)_ = 11.113, *p* < 0.001; Figure 2C). *Post hoc* comparisons showed that KET-SAL animals committed significantly more errors than all other treatment groups. These errors were further broken down into perseverative, regressive and never reinforced errors. While there was not a significant effect of treatment on perseverative errors (Persev: *F*_(3,32)_ = 0.834, *p* = 0.485; Figure 2D), there was a significant effect on both regressive and never reinforced errors (Reg: *F*_(3,32)_ = 8.610, *p* < 0.001; NR: *F*_(3,32)_ = 9.592, *p* < 0.001; Figure 2D). The majority of animals required only one probe trial to reach criterion and accordingly there were no significant differences in the number of probe trials between treatment groups during either Response Discrimination (*F*_(3,32)_ = 0.620, *p* = 0.607) or Shift-to-Visual-Cue Discrimination (*F*_(3,32)_ = 0.658, *p* = 0.584; data not shown). Taken together, these results show that ketamine treatment did not interfere with the animals’ ability to acquire the first strategy, but resulted in a selective impairment in performing the shift to a visual cue-based strategy. Importantly, animals treated with NAC exhibit normal cognitive flexibility, learning the shift to the visual cue strategy at the same rate as controls.

### Delayed Spontaneous Alternation

**Figure 3:**
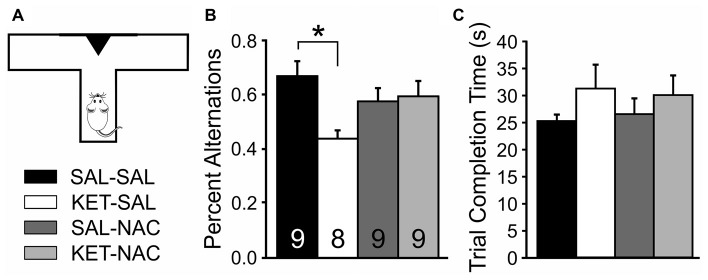
NAC preserves normal foraging behavior, which is impaired following developmental ketamine treatment. **(A)** Animals were placed in the stem arm of a modified T-maze and allowed to enter the left or right arm, respectively. After the choice, animals were confined to that arm for 30 s and then placed back immediately into the stem arm for a second free-choice trial. If the animal selected the arm opposite to its initial choice this was considered a spontaneous alternation. Pairs of free-choice runs were repeated for a total of six trials, with 20 min separating each trial. **(B)** KET-SAL animals performed significantly less spontaneous alternations compared to the SAL-SAL animals while NAC-treated animals did not perform significantly different from controls. This suggests that ketamine treatment disrupts short term memory required for normal foraging behavior, and that this is ameliorated by NAC treatment. **(C)** All treatment groups completed the trials in a comparable amount of time. Significance is indicated as * for *P* < 0.05, following Bonferroni correction.

When exploring their environment, drug-naïve rodents show a strong tendency to alternate entries into the arms of a maze. In order to measure treatment-induced changes in spatial learning and short term memory, we measured the frequency of spontaneous alternations between entries into a left or right goal-arm during free-choice trials in a T-maze (Figure 3). A one-way ANOVA showed a significant effect of drug treatment on spontaneous alternation (*F*_(3,31)_ = 3.506, *p* = 0.027). *Post hoc* comparisons revealed that KET-SAL animals showed significantly fewer alternations than animals in the SAL-SAL group (Figure 3B). In contrast, mice in the KET-NAC and SAL-NAC groups showed similar patterns of alternation as the SAL-SAL group, indicating that co-administration of NAC was able to protect against ketamine-induced deficits in spatial learning and memory. There were no differences between treatment groups in the average time to completion (*F*_(3,31)_ = 0.781, *p* = 0.514; Figure 3C), making it unlikely that ketamine-induced differences in the alternation pattern reflect an unspecific deficit in overall activity.

### Novel Object Recognition

**Figure 4:**
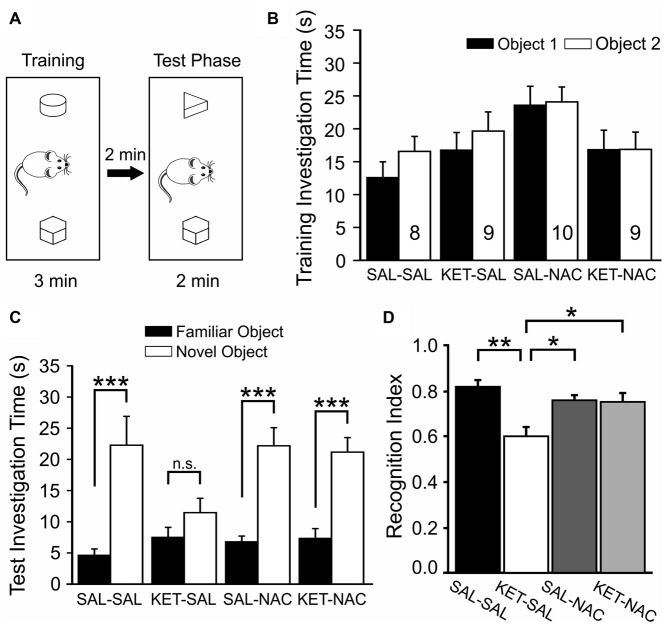
NAC treatment protects against deficits in novel object recognition induced by ketamine treatment. **(A)** Mice were placed in an open box and allowed to investigate two wooden objects for 3 min before they were placed back into their home cage for 2 min. One of the two objects was replaced with a novel object and animals were allowed to explore both objects for an additional 2 min. **(B)** Total investigation time during the training phase did not differ significantly between treatment groups, and animals showed no inherent preferences for any one object. **(C)** During the test phase, animals in all groups spent significantly more time with the novel object, with the exception of mice in the KET-SAL group, which showed no significant preference for the novel object. **(D)** A Recognition Index was calculated by dividing the amount of time spent with the novel object over the total time spent investigating both objects. Ketamine-treated animals scored lower recognition indices, suggesting a deficit in their ability to differentiate between familiar and novel objects. Ketamine-treated animals that also received NAC showed recognition indices comparable to controls. Significance is indicated as * for *P* < 0.05, ** for *P* < 0.01, and *** for *P* < 0.001, following Bonferroni correction.

Next, we tested how developmental ketamine treatment affects the ability of mice to distinguish between a familiar and a novel object (Figure 4). We first introduced animals to two objects during the training phase and scored the investigation time for each of the two objects. A two-way mixed ANOVA with treatment as the between-subjects factor and the two objects as the within-subjects factor revealed no significant difference in investigation time between treatment groups (*F*_(3,32)_ = 2.588, *p* = 0.070) and no difference between objects (*F*_(1,32)_ = 3.945, *p* = 0.056; Figure 4B). After a 2 min delay, animals were then exposed to one of the familiar objects and a novel object, and we again scored investigation times for each of the two objects. As expected, there was a significant difference in investigation time between the familiar and novel objects (*F*_(1,32)_ = 81.364, *p* < 0.001l) suggesting an overall preference for the novel object across all groups. However, this was qualified by an interaction between treatment group and object preference (*F*_(3,32)_ = 4.545, *p* = 0.009). Further analysis of simple main effects revealed that this interaction was due to the KET-SAL group, which showed no significant preference for the novel object (Figure 4C). For each animal we also calculated a recognition index, which normalized the novel investigation time to a percent of the total investigation time. A one-way ANOVA revealed a significant effect of treatment on this recognition index (*F*_(3,32)_ = 7.306, *p* = 0.001; Figure 4D). *Post hoc* comparisons revealed that KET-SAL animals showed significantly lower recognition indices than animals in the other three treatment groups. On the other hand the recognition index of NAC-treated animals did not differ significantly from that of mice in the SAL-SAL control group, indicating that NAC was able to prevent deficits in novel object recognition that result from developmental ketamine-treatment.

### Social Interaction

**Figure 5:**
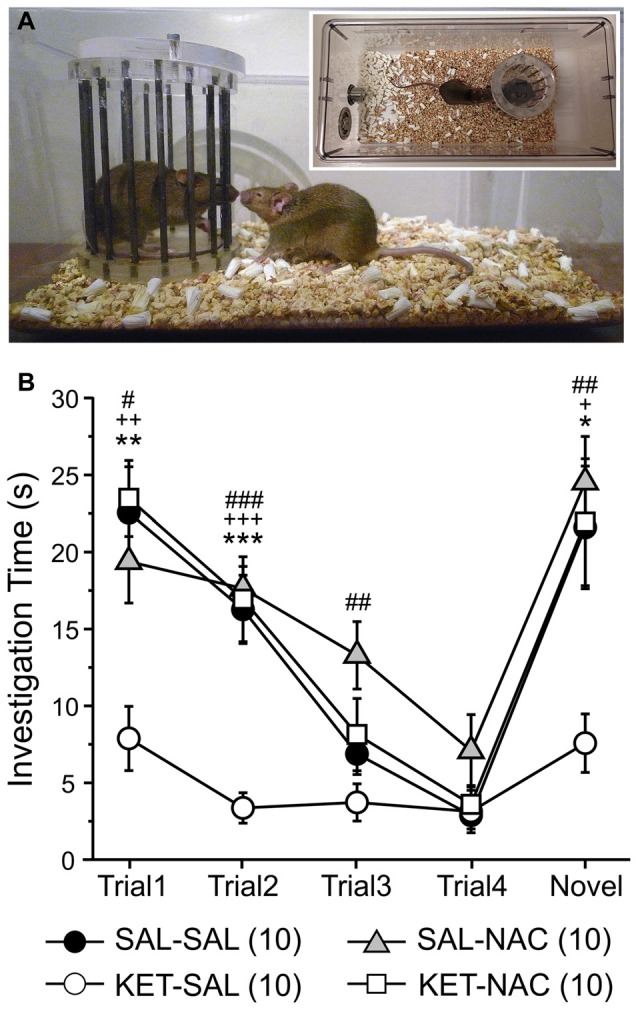
Ketamine treatment disrupts normal social investigation patterns, which is prevented by concomitant NAC treatment. **(A)** Stimulus mice were introduced repeatedly into the home cage of experimental mice in order to test social memory and interaction patterns. Four exposures to the same stimulus mouse (Trials 1–4) were followed by presentation of a novel stimulus mouse (Trial 5). **(B)** Both saline control (SAL-SAL) and NAC-treated animals (SAL-NAC, KET-NAC) showed a progressive decrease in investigation time with repeated presentation of the initial stimulus mouse. Investigation times increase again in Trial 5 upon presentation of the novel stimulus mouse. In contrast, ketamine-treated animals (KET-SAL) show consistently low investigation times throughout all 5 trials.Significance is indicated as * for *P* < 0.05 SAL-SAL vs. KET-SAL; ** for *P* < 0.01 SAL-SAL vs. KET-SAL; *** for *P* < 0.001 SAL-SAL vs. KET-SAL; ^+^ for *P* < 0.05 KET-NAC vs. KET-SAL; ^++^ for *P* < 0.01 KET-NAC vs. KET-SAL; ^+++^ for *P* < 0.001 KET-NAC vs. KET-SAL; ^#^ for *P* < 0.05 SAL-NAC vs. KET-SAL; ^##^ for *P* < 0.01 SAL-NAC vs. KET-SAL; ^###^ for *P* < 0.001 KET-NAC vs. KET-SAL, following Bonferroni correction.

Next, we used a social interaction task to assess sociability and preference for social novelty, which may be relevant to negative symptoms of schizophrenia and possibly to some features of depression. We have previously shown that perinatal ketamine treatment induces deficits in social interaction, leading to greatly reduced interaction times of the KET-SAL mice with the stimulus mice across all exposures (Jeevakumar et al., 2015). Here we replicate these findings and examine whether N-acetylcysteine treatment can prevent these social deficits (Figure 5). A two-way mixed ANOVA, with drug-treatment as the between-groups factor and trial number as the within-group factor revealed a significant main effect of treatment (*F*_(3,36)_ = 12.965, *p* < 0.001; Figure 5B) and a significant interaction between trial number and treatment (*F*_(12,144)_ = 2.709, *p* = 0.003) on social interaction. Compared to animals in the other three treatment groups ketamine-treated mice interacted significantly less with the first stimulus mouse during trials 1 and 2. With repeated exposures of a now familiar mouse interaction times continued to decease in the other three treatment groups, and by trial 3 and 4 interaction times were similar across all groups. However, upon exposure to a novel stimulus mouse, interaction times significantly increased again in all treatment groups, except the KET-SAL group, indicating deficits in social interaction in ketamine-treated animals (Figure 4B). Importantly, KET-NAC animals showed normal patterns and durations of interaction times that were indistinguishable from SAL-SAL controls, indicating that NAC prevented the development of ketamine-induced deficits in social interaction.

### Prepulse Inhibition of the Acoustic Startle Response

**Figure 6:**
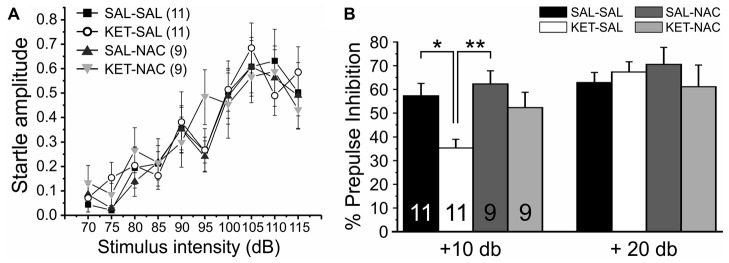
Perinatal ketamine treatment reduces PPI of the acoustic startle response in adult mice. **(A)** Baseline startle responses for the four treatment groups over a range of startle pulse intensities (75 dB–115 dB). There were no significant differences in the startle response between groups. **(B)** Prepulse inhibition. Mice were presented with 20 startle tones that were preceded by prepulses of either 75 dB (10 dB over background noise) or 85 dB (20 dB over background noise), respectively. Perinatal ketamine treatment impaired PPI under the 10 dB prepulse condition, but not the 20 dB condition. Co-administration of NAC with ketamine failed to fully prevent the ketamine-induced reduction in PPI. Significance is indicated as * for *P* < 0.05 and ** for *P* < 0.01, following Bonferroni correction.

Prepulse inhibition (PPI) of the startle response is a measure of sensorimotor gating that is reduced in patients with schizophrenia (Braff and Geyer, 1990) and various rodent models of the disease (Lipska et al., 1995; Young et al., 2009; Powell et al., 2012b), including models that induce NMDAR dysfunction during development (Wedzony et al., 2008; Lyall et al., 2009). In humans, PPI deficits correlate with distractibility (Karper et al., 1996), and with quantitative measures of thought disorder (Perry and Braff, 1994). In order to test the effects of perinatal ketamine treatment and NAC on sensorimotor gating, we first measured startle responses over a range of increasing startle pulse intensities to ensure that ketamine or NAC treatment did not affect the baseline startle response. Then we tested PPI under two different prepulse conditions (10 or 20 dB over background noise). There were no significant differences in either the normalized input-output curve of startle responses (*F*_(3,36)_ = 0.287, *p* = 0.835; Figure 6A) or baseline startle responses to the startle pulse alone during PPI (*F*_(3,36)_ = 1.412, *p* = 0.255), indicating that animals in all treatment groups startled to the same extent. In contrast, a one-way ANOVA with PPI as the dependent variable and treatment as the between-groups factor revealed a significant effect of treatment on PPI for the 10 dB condition (*F*_(3,36)_ = 5.298, *p* = 0.004). Bonferroni corrected *post hoc* tests revealed a significant effect between SAL-SAL and KET-SAL, as well as SAL-NAC and KET-SAL animals (Figure 6B). However, even though there was clear trend, the KET-NAC and KET-SAL groups did not differ statistically, indicating that NAC failed to fully protect against the ketamine-induced deficits in PPI. When the prepulse was more salient (20 dB condition), mice in all groups exhibited comparable PPI (*F*_(3,36)_ = 0.449, *p* = 0.719). Our data suggests that perinatal ketamine treatment results in a long-lasting deficit in PPI, which can be partly recovered by concomitant NAC application.

### Amphetamine-Induced Locomotor Activity

**Figure 7:**
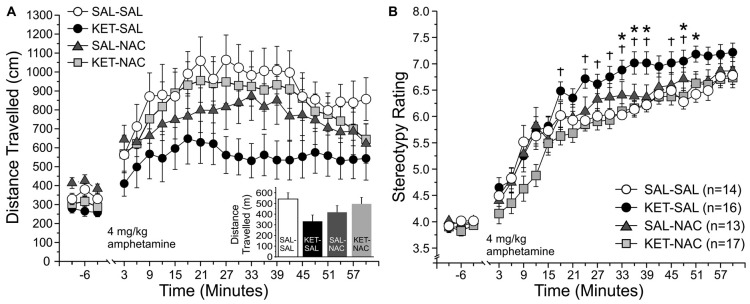
NAC treatment prevents ketamine-induced increases in stereotypic locomotor behavior in response to an acute injection of amphetamine. Animals were placed in an open field and baseline activity was recorded for 30 min. Mice were then injected with 4 mg/kg d-amphetamine and their locomotor and stereotypic behavior was recorded for an additional 60 min. **(A)** Total distance traveled. Movement data is averaged into 3 min bins. The last 9 min of baseline activity and all 20 bins following the amphetamine injection were selected for analysis. Total distance traveled did not significantly differ between treatment groups during either the baseline- or post-injection periods; however, KET-SAL animals showed a trend to reduced movement during the post-injection period. **(B)** Analysis of stereotypic behavior on a scale from 1 to 9 revealed that KET-SAL animals showed significantly more stereotypic behaviors than SAL-SAL or KET-NAC animals at several points following amphetamine injection (but not during baseline), which may explain the reductions in total distance traveled. Significance is indicated as * for *P* < 0.05 SAL-SAL vs. KET-SAL, and ^†^ for *p* < 0.05 KET-NAC vs. KET-SAL.

Dysregulation of subcortical dopamine function plays an important role in the pathophysiology of schizophrenia, and psychostimulants like amphetamine, which enhance dopamine transmission, can exacerbate psychotic symptoms in schizophrenic subjects (Breier et al., 1997; Abi-Dargham et al., 1998). For this reason, the effects of psychotropic drugs on locomotor activity have widely been used to model positive symptoms of schizophrenia in rodents (Geyer and Markou, 1995; van den Buuse, 2010). Here we tested whether treatment with NAC can prevent the increased sensitivity to d-amphetamine that is seen following perinatal ketamine treatment (Jeevakumar et al., 2015). We measured both changes in overall locomotor activity (Figure 7A), as well as stereotypic behavior (Figure 7B). We first assessed overall locomotor activity as the total distance traveled. A two-way mixed ANOVA with total distance traveled as the dependent variable, drug treatment as the between-groups factor, and time as the within-groups factor revealed a significant main effect of time (*F*_(22,1034)_ = 26.989, *p* < 0.001) on overall locomotor activity; however, we found no significant main effect of treatment (*F*_(3,47)_ = 1.843, *p* = 0.152) and no interaction effect of time and treatment (*F*_(66,1034)_ = 1.134, *p* = 0.223). Although there was no significant main effect of treatment on total distance traveled across the four experimental groups, KET-SAL animals showed a trend towards reduced amphetamine-induced locomotor activity. Amphetamine-induced reductions in locomotor behavior may reflect increased repetitive movement in place, characterized by nearly continuous repetitive head movements, forelimb movements, sniffing, licking or biting (Ellinwood and Balster, 1974; Robinson and Becker, 1986). In order to explore this possibility, we rated stereotypic movements on a scale from 1 to 9 (Ellinwood and Balster, 1974) across the same time bins shown in Figure 6A. We found a significant main effect of time (*F*_(22,1232)_ = 158.616, *p* < 0.001), as well as a significant main effect of drug treatment (*F*_(3,56)_ = 4.844, *p* = 0.005) on stereotypic behavior. Both of these main effects were qualified by a significant interaction effect of time and treatment (*F*_(66,1232)_ = 1.314, *p* = 0.050). Further analysis of simple main effects revealed that KET-SAL animals exhibited increased stereotypy compared to SAL-SAL and KET-NAC animals across several time points following amphetamine injection, without significant differences in baseline activity (Figure 7B). These results indicate that developmental ketamine administration increases the sensitivity to psychostimulants that stimulate the subcortical dopamine system, leading to increased stereotypic motor behavior in mice. Co-treatment with NAC prevented emergence of these symptoms.

## Discussion

Early-life stressors are associated with neuropsychiatric vulnerability and an increased risk of several mental illnesses, including schizophrenia (Agid et al., 1999; Powell, 2010; Young et al., 2012). Patients with schizophrenia suffer from deficits in working memory, attention and cognitive flexibility, as well as social cognition and interaction (Nuechterlein et al., 2004). Cognitive deficits are the major determinant of long-term functional outcome in schizophrenic patients (Green, 1996). Because they are only poorly responsive to current treatments, there is a renewed focus on defining and treating cognitive deficits (Green et al., 2004; Nuechterlein et al., 2004). In rodents, perinatal exposure to NMDA receptor-antagonists causes deficits in adulthood in a number of tasks that mimic cognitive, as well as positive and negative symptoms of schizophrenia (Wang et al., 2003; Stefani and Moghaddam, 2005; Broberg et al., 2008; Jeevakumar et al., 2015). Here we show that co-treatment with the GSH precursor N-acetyl cysteine can prevent development of several of these complex behavioral deficits.

So-called “executive functions” comprise a set of cognitive processes that include attentional control, working memory, cognitive inhibition, response inhibition and cognitive flexibility. Executive functions enable adaptations in behavioral responses that vary based on the context of a situation. Deficits in executive function are most strongly related to dysfunction of the PFC. Individuals with schizophrenia or with damage to the PFC exhibit impairments in tasks that assess cognitive flexibility (Addington and Addington, 1997; Pantelis et al., 1999) such as the Wisconsin Card Sorting Test (WCST), which requires engagement of multiple cognitive domains, including working memory, reversal learning, attentional set-shifting and sustained attention (Eling et al., 2008). An animal equivalent of the WCST with high validity for executive functions is the attentional set-shifting task (Birrell and Brown, 2000; Young et al., 2009, 2010), which requires animals to perform one or several intra-dimensional set shifts, followed by an extra-dimensional set shift. Other tasks which more broadly test the concept of “cognitive flexibility” (including the Response Discrimination and Shift-to-Visual-Cue Discrimination used here) typically require rodents to acquire a new strategy while inhibiting the use of a previously reinforced strategy; however, without first establishing a cognitive “set” or comparing intra- and extra-dimensional shifts (Ragozzino, 2002; Stefani and Moghaddam, 2005; Floresco et al., 2006; Jeevakumar et al., 2015). Pharmacological blockade of NMDARs induces deficits in these tests of cognitive flexibility, both when antagonists are administered acutely in adult animals (Stefani et al., 2003; Neill et al., 2010; Nikiforuk et al., 2010), or briefly during development (Stefani and Moghaddam, 2005; Broberg et al., 2008; Jeevakumar et al., 2015). Manipulations during development do not affect the acquisition of the original strategy, but impair the shift to a new rule (Stefani and Moghaddam, 2005; Jeevakumar et al., 2015). Here we find that ketamine-treated animals committed more regressive and never-reinforced errors during the shift than control or NAC-treated animals. However, ketamine-treatment did not significantly increase the number of perseverative errors, which traditionally are seen as the most direct indicator of impaired executive functions (Eling et al., 2008). This result is in contrast with our own previous data (Jeevakumar et al., 2015) where we found an increase in perseverative errors in ketamine-treated animals. The reason for this discrepancy are not immediately clear, but probably reflects the fact that our definition of what constitutes a perseverative error (three out of four responses within a block of trials) allows for only a small number of perseverative errors, which may obscure relatively small effects across different cohorts of animals. In rodents, deficits in cognitive flexibility indicate disruptions in medial PFC function (Birrell and Brown, 2000; Floresco et al., 2009). The impairments in cognitive flexibility caused by perinatal NMDAR blockade are associated with persistent alterations in glutamatergic transmission and interneuron function in the PFC (Jones et al., 2014; Jeevakumar et al., 2015; Jeevakumar and Kroener, 2016). Several lines of evidence suggest that alterations in oxidative defense mechanisms result in prolonged oxidative insult on GABAergic neurons (Behrens et al., 2007; Hardingham and Do, 2016) and that these changes affect glutamate release in the PFC (Sorce et al., 2010; Jeevakumar and Kroener, 2016). These changes are thought to recapitulate important aspects of dysfunctions in glutamate metabolism and release, as well as deficits in antioxidant defense mechanisms that have been reported in the PFC of patients with schizophrenia (Do et al., 2000; Gawryluk et al., 2011; Flatow et al., 2013; Hardingham and Do, 2016). Our results suggest that antioxidant treatment with NAC can counteract the effects of perinatal NMDAR antagonism to protect complex prefrontal cortical functions.

Patients with schizophrenia also exhibit impairments in declarative memory (Weiss and Heckers, 2001; Stone and Hsi, 2011) and the ability to distinguish objects as novel or familiar (Heckers et al., 2000; Bozikas et al., 2006). In rodent models of schizophrenia, the novel object recognition task has been used as an ethologically relevant paradigm to study visual episodic memory and to assess changes in task performance that result from pharmacological intervention (Grayson et al., 2007; Venâncio et al., 2011; Rajagopal et al., 2014). The task uses the innate tendency of rodents to investigate novel objects as a way to assess recognition memory. Impaired performance in this task is thought to indicate dysregulated interactions between the PFC and the hippocampus (Weiss and Heckers, 2001). The medial PFC is involved in recency discrimination and may consolidate and control the retrieval of information from short-term episodic memory (Morici et al., 2015) and interact with the perirhinal cortex to support recognition memory (Barker et al., 2007). Patients with schizophrenia show lower NMDAR binding in the dentate gyrus of the hippocampus (Law and Deakin, 2001). Both acute (de Lima et al., 2005; Grayson et al., 2007; Pitsikas et al., 2008; Nikiforuk et al., 2013) and subchronic NMDAR blockade in adult animals (Hashimoto et al., 2007; Nagai et al., 2009), as well as transient NMDAR antagonism during development (Jeevakumar et al., 2015) lead to deficits in recognition memory. Dysregulation of GSH impairs parvalbumin-positive interneurons and gamma oscillations in the hippocampus, leading to impairments in spatial memory (Steullet et al., 2010), which can be reversed by chronic NAC treatment (Choy et al., 2010). Our data suggest that NAC can also prevent the emergence of ketamine-induced impairments in recognition memory.

Perinatal ketamine treatment also reduced spontaneous alternation behavior. Impaired performance in the Spontaneous Alternation task can result from lesions of the medial PFC or corticolimbic pathways that lead to behavioral disinhibition (Lalonde, 2002), and lower alternation scores have been taken to indicate impairments in spatial working memory (Stefani and Moghaddam, 2005; Castañé et al., 2015; Grayson et al., 2016). Subchronic (but not acute) PCP treatment in adult animals impairs performance in spontaneous alternation (Castañé et al., 2015; Grayson et al., 2016). Similarly, perinatal MK-801 treatment in rats also leads to impaired spontaneous alternation in adults (Stefani and Moghaddam, 2005). We show that co-treatment with NAC can increase the number of alternations in mice treated perinatally with ketamine, further indicating that antioxidant treatment during a critical period can reduce oxidative stress in corticolimbic networks that regulate exploratory behavior and spatial working memory. Interestingly, we observed changes in spontaneous alternation behavior in KET-SAL animals, even though ketamine treatment did not affect acquisition of the egocentric response strategy during the Response Discrimination phase of our test of cognitive flexibility, which also requires animals to remember their recent choice (see Figure 2B). This discrepancy most likely reflects differences in the memory requirements of the two tasks, as well as differences in the motivation and attentional state of the animals. In the Spontaneous Alternation task, each individual trial consisted of two choices that were separated by 30 s and each of the six trials were separated by 20 min intervals. In contrast, during Response Discrimination training animals were placed back into the stem arm immediately after they had made a choice and consumed the food reward (if available). These differences in the timing of choice-runs are likely to affect short-term memory requirements. Furthermore, in contrast to animals that performed spontaneous, unreinforced alternations, animals that learned the Response Discrimination were food-restricted and well trained to actively search for food rewards, which should greatly affect their motivation and attention, and should ultimately affect short-term memory performance.

Asociality and social withdrawal are distinct negative symptoms of schizophrenia and are one of the earliest symptoms observed (McClellan et al., 2003; Johnstone et al., 2005). In both prospective and retrospective studies impaired social function and isolation have been shown to precede the onset of psychotic symptoms of schizophrenia (Häfner et al., 1999; Møller and Husby, 2000; Addington et al., 2008) and they are a common risk factor for the development of psychosis in high-risk patients (Cannon et al., 2008). The social interaction task is a comprehensive way to assess recognition memory in rodents because it incorporates multiple sensory modalities such as olfactory, tactile, auditory and visual processing, all of which are necessary for social interaction (Moy et al., 2004). Administration of ketamine or PCP in adult animals (Sams-Dodd, 1998; Wang et al., 2007; Nikiforuk et al., 2013), as well as perinatal treatment with PCP (Terranova et al., 2005; Harich et al., 2007) have been shown to produce impairments in novelty discrimination in a social recognition task. Here we replicated our previous findings (Jeevakumar et al., 2015) to show that ketamine-treated animals overall interact significantly less with the stimulus mouse, and also display no signs of novelty discrimination when a new stimulus mouse is introduced. Perinatal KET treatment therefore induces persistent changes in behavior that mimic important aspects of the negative symptoms of schizophrenia. Animals treated with NAC showed improved social interaction and novelty discrimination, as well as reduced social withdrawal, suggesting that treatment with NAC has protective effects in those brain regions associated with social cognition that are affected by perinatal NMDAR blockade.

Measures of drug-induced locomotor activity and stereotypy are widely used to probe the functional integrity of the dopamine and glutamate systems (Segal et al., 1981; Swerdlow et al., 1986). Because patients with schizophrenia show exaggerated dopamine release and exacerbated symptoms in response to amphetamine (Laruelle et al., 1996), measuring changes in amphetamine-induced locomotor activity in animals has proved helpful to identify drugs that can treat the positive symptoms of schizophrenia. In animals that were treated perinatally with ketamine, the relatively high dose of amphetamine that we tested here reduced the total distance traveled, resulting from a significant increase in stereotypical behavior. Co-treatment with NAC reduced stereotypical behavior in ketamine mice and increased the total distance traveled to values seen in saline-treated control animals. This indicates that antioxidant treatment can also protect against changes in the subcortical dopamine system and the striatum that may underlie the positive symptoms of schizophrenia (Howes and Kapur, 2009).

Dopaminergic modulation of corticolimbic circuits also affects sensorimotor gating (Swerdlow et al., 2001). Sensorimotor gating describes the ability of a weak sensory event to inhibit the motor response to a strong sensory stimulus, and is thought to rely on central inhibitory mechanisms that strongly influence the structure and cohesiveness of thought (Swerdlow et al., 2001). PPI of the startle response is the most commonly used measure of such “pre-attentional” sensory processing mechanisms. In humans, deficits in PPI correlate with distractibility (Karper et al., 1996), and with quantitative measures of thought disorder (Perry and Braff, 1994; Perry et al., 1999); however, PPI is not unique to schizophrenia and the relationship between PPI and cognitive functions is not clear (reviewed in Young et al., 2009). Using perinatal ketamine application, we found deficits in PPI, similar to those reported previously following subchronic blockade of NMDARs during development in rats (Wedzony et al., 2008; Lyall et al., 2009). These deficits in PPI thus lend further support to the notion that perinatal ketamine application mimics certain relevant aspects of schizophrenia. Previous work using the neonatal ventral hippocampal lesion model of schizophrenia has already shown that deficits in PPI are improved in adult rodents when NAC is administered during development (Cabungcal et al., 2014). In our data, NAC co-treatment also ameliorated ketamine-induced deficits in PPI; however, these effects did not attain significance in our relatively small sample.

Taken together, our data suggest that antioxidant treatment during a critical developmental period can prevent a wide range of cognitive and sensorimotor deficits in a rodent model of NMDAR dysfunction in schizophrenia. Current treatments for schizophrenia, which primarily work through D2 dopamine receptor antagonism, are most effective for the positive symptoms, but show little efficacy for the negative symptoms or cognitive deficits of the disease (Mailman and Murthy, 2010). Oxidative stress is an important factor in the pathophysiology of the disease. Both peripheral tissue and neurons in the CNS of schizophrenic patients show oxidative stress and abnormal levels of antioxidants, including GSH, which provides endogenous protection against ROS and RNS (Gawryluk et al., 2011; Do et al., 2015). Deregulation of ROS is correlated with negative symptoms and cognitive deficits of the disease (Matsuzawa and Hashimoto, 2011; Zhang et al., 2012; Rajasekaran et al., 2015). For these reasons antioxidants hold considerable potential for the treatment or prevention of pathologies in schizophrenia (Do et al., 2015).

Supplementation with NAC has previously also been shown to prevent changes in the expression of parvalbumin, a marker of fast-spiking GABAergic neurons, which occur in several animal models of schizophrenia (Cabungcal et al., 2013, 2014), and which are a hallmark of morphological changes seen in postmortem brains of schizophrenic patients (Lewis et al., 2012). The reduction in parvalbumin-positive interneurons is thought to be related to NMDA receptor dysfunction (Bitanihirwe and Woo, 2011; Jeevakumar and Kroener, 2016; Kantrowitz et al., 2016). NMDAR antagonists trigger a rapid increase in ROS *in vitro* (Xia et al., 2002), and *in vivo* (Zuo et al., 2007; Powell et al., 2012a). The effects of ketamine are related to an increase in the proinflammatory cytokine interleukin-6 and activation of the superoxide-producing enzyme NADPH-oxidase (NOX) which is a major source of ROS and affects glutamate release in the PFC (Behrens et al., 2007; Sorce et al., 2010; Powell et al., 2012a). In addition, there exists crosstalk between NOX and mitochondrial ROS that may cause severe mitochondrial dysfunction, leading to further intracellular redox imbalance and calcium perturbations (Wenzel et al., 2008; Kröller-Schön et al., 2014). NOX-dependent oxidative mechanisms have been implicated in schizophrenia-like behaviors in different animal models (Schiavone et al., 2009; Cabungcal et al., 2014). Decreased levels of GSH are found in the cerebrospinal fluid, as well as postmortem tissue of drug-naive schizophrenia patients (Do et al., 2000, 2009; Yao et al., 2006). Polymorphisms in genes that code for enzymes involved in the synthesis of GSH have been linked to risk for developing schizophrenia (Tosic et al., 2006; Gysin et al., 2007). Disrupting GSH synthesis in mice alters the morphology and function of hippocampal PV interneurons (Steullet et al., 2010). Cysteine is the rate-limiting substrate for the synthesis of GSH and thus supplementation of NAC is thought to be an avenue to promote the resynthesis of GSH to neutralize free radicals. Because of its anti-oxidative and anti-inflammatory properties NAC has been successfully applied to different models of schizophrenia (Lutgen et al., 2013; Cabungcal et al., 2014). In a small number of studies NAC has been used as an add-on treatment in schizophrenic patients where it improved negative symptoms, working memory and global functioning (Berk et al., 2008; Lavoie et al., 2008; Farokhnia et al., 2013; Rapado-Castro et al., 2017). The protracted progression to psychosis represents both a window of vulnerability and opportunity for therapeutic intervention (Do et al., 2015). Prophylactic treatments aim to affect the prodromal phase of schizophrenia and to stop the progression to psychosis. NAC is generally considered well tolerated and shows few side effects (Berk et al., 2013), and therefore NAC could provide a low-risk treatment to halt the progression of schizophrenia during adolescence. However, antioxidants can perform differently depending on the redox status of the organism and the cellular milieu, so that they act either as an antioxidant or as a pro-oxidant (Harvey et al., 2008; Möller et al., 2015). Therefore, in order to maximize the utility of antioxidants as a treatment for early intervention, reliable biomarkers are needed that allow to identify presymptomatic oxidative stress during the prodromal stages of schizophrenia. In addition, dose-finding studies are needed to determine the optimal dose of NAC, as well as a profile of efficacy for the various symptoms of the disease. Finally, it must be noted that all our experiments were performed in male mice. However, there are well-known sex-differences in the onset and progression of schizophrenia (with first-time hospitalizations occurring at a younger age in men than in women), as well as sex-specific differences in the performance of most of the cognitive tasks that we tested here in both animals and humans (for review see Hill, 2016; Leger and Neill, 2016). Therefore, future studies must also compare the effectiveness of NAC as a potential treatment in female populations.

## Author Contributions

AP and HED contributed equally to this work, participating in all steps of this investigation and the writing of the manuscript. SB, AP, CD, AB and GP performed additional experiments and analyzed data. SK designed all experiments, performed analysis and wrote the manuscript.

## Conflict of Interest Statement

The authors declare that the research was conducted in the absence of any commercial or financial relationships that could be construed as a potential conflict of interest.

## References

[B1] Abi-Dargham A. Gil R. Krystal J. Baldwin R. M. Seibyl J. P. Bowers M.. (1998). Increased striatal dopamine transmission in schizophrenia: confirmation in a second cohort. Am. J. Psychiatry 155, 761–767. 9619147 10.1176/ajp.155.6.761

[B2] Addington J. Addington D. (1997). Attentional vulnerability indicators in schizophrenia and bipolar disorder. Schizophr. Res. 23, 197–204. 10.1016/s0920-9964(96)00105-3 9075297

[B3] Addington J. Penn D. Woods S. W. Addington D. Perkins D. O. (2008). Social functioning in individuals at clinical high risk for psychosis. Schizophr. Res. 99, 119–124. 10.1016/j.schres.2007.10.001 18023329 PMC2292799

[B4] Agid O. Shapira B. Zislin J. Ritsner M. Hanin B. Murad H.. (1999). Environment and vulnerability to major psychiatric illness: a case control study of early parental loss in major depression, bipolar disorder and schizophrenia. Mol. Psychiatry 4, 163–172. 10.1038/sj.mp.4000473 10208448

[B5] Almaguer-Melian W. Cruz-Aguado R. Bergado J. A. (2000). Synaptic plasticity is impaired in rats with a low glutathione content. Synapse 38, 369–374. 10.1002/1098-2396(20001215)38:4<369::AID-SYN1>3.0.CO;2-Q 11044883

[B6] Amann L. C. Gandal M. J. Halene T. B. Ehrlichman R. S. White S. L. McCarren H. S.. (2010). Mouse behavioral endophenotypes for schizophrenia. Brain Res. Bull. 83, 147–161. 10.1016/j.brainresbull.2010.04.008 20433908

[B8] Amitai N. Semenova S. Markou A. (2007). Cognitive-disruptive effects of the psychotomimetic phencyclidine and attenuation by atypical antipsychotic medications in rats. Psychopharmacology 193, 521–537. 10.1007/s00213-007-0808-x 17497138

[B9] Barker G. R. I. Bird F. Alexander V. Warburton E. C. (2007). Recognition memory for objects, place, and temporal order: a disconnection analysis of the role of the medial prefrontal cortex and perirhinal cortex. J. Neurosci. 27, 2948–2957. 10.1523/JNEUROSCI.5289-06.2007 17360918 PMC6672574

[B10] Behrens M. M. Ali S. S. Dao D. N. Lucero J. Shekhtman G. Quick K. L.. (2007). Ketamine-induced loss of phenotype of fast-spiking interneurons is mediated by NADPH-oxidase. Science 318, 1645–1647. 10.1126/science.1148045 18063801

[B11] Berk M. Copolov D. Dean O. Lu K. Jeavons S. Schapkaitz I.. (2008). N-acetyl cysteine as a glutathione precursor for schizophrenia—a double-blind, randomized, placebo-controlled trial. Biol. Psychiatry 64, 361–368. 10.1016/j.biopsych.2008.03.004 18436195

[B12] Berk M. Malhi G. S. Gray L. J. Dean O. M. (2013). The promise of N-acetylcysteine in neuropsychiatry. Trends Pharm. Sci. 34, 167–177. 10.1016/j.tips.2013.01.001 23369637

[B13] Birrell J. M. Brown V. J. (2000). Medial frontal cortex mediates perceptual attentional set shifting in the rat. J. Neurosci. 20, 4320–4324. 10818167 10.1523/JNEUROSCI.20-11-04320.2000 PMC6772641

[B14] Bitanihirwe B. K. Y. Woo T. U. W. (2011). Oxidative stress in schizophrenia: an integrated approach. Neurosci. Biobehav. Rev. 35, 878–893. 10.1016/j.neubiorev.2010.10.008 20974172 PMC3021756

[B15] Bozikas V. P. Kosmidis M. H. Kiosseoglou G. Karavatos A. (2006). Neuropsychological profile of cognitively impaired patients with schizophrenia. Compr. Psychiatry 47, 136–143. 10.1016/j.comppsych.2005.05.002 16490572

[B16] Braff D. L. Geyer M. A. (1990). Sensorimotor gating and schizophrenia. Human and animal model studies. Arch. Gen. Psychiatry 47, 181–188. 10.1001/archpsyc.1990.01810140081011 2405807

[B17] Breier A. Su T. P. Saunders R. Carson R. E. Kolachana B. S. de Bartolomeis A.. (1997). Schizophrenia is associated with elevated amphetamine-induced synaptic dopamine concentrations: evidence from a novel positron emission tomography method. Proc. Natl. Acad. Sci. U S A 94, 2569–2574. 10.1073/pnas.94.6.2569 9122236 PMC20129

[B18] Broberg B. V. Dias R. Glenthøj B. Y. Olsen C. K. (2008). Evaluation of a neurodevelopmental model of schizophrenia—Early postnatal PCP treatment in attentional set-shifting. Behav. Brain Res. 190, 160–163. 10.1016/j.bbr.2008.02.020 18367258

[B19] Cabungcal J.-H. Counotte D. S. Lewis E. M. Tejeda H. A. Piantadosi P. Pollock C.. (2014). Juvenile antioxidant treatment prevents adult deficits in a developmental model of schizophrenia. Neuron 83, 1073–1084. 10.1016/j.neuron.2014.07.028 25132466 PMC4418441

[B20] Cabungcal J.-H. Steullet P. Kraftsik R. Cuenod M. Do K. Q. (2013). Early-life insults impair parvalbumin interneurons via oxidative stress: reversal by *N*-acetylcysteine. Biol. Psychiatry 73, 574–582. 10.1016/j.biopsych.2012.09.020 23140664

[B21] Cannon T. D. Cadenhead K. Cornblatt B. Woods S. W. Addington J. Walker E.. (2008). Prediction of psychosis in youth at high clinical risk. Arch. Gen. Psychiatry 65, 28–37. 10.1001/archgenpsychiatry.2007.3 18180426 PMC3065347

[B22] Castañé A. Santana N. Artigas F. (2015). PCP-based mice models of schizophrenia: differential behavioral, neurochemical and cellular effects of acute and subchronic treatments. Psychopharmacology 232, 4085–4097. 10.1007/s00213-015-3946-6 25943167

[B23] Chatterjee M. Ganguly S. Srivastava M. Palit G. (2011). Effect of chronic versus acute ketamine administration and its withdrawal effect on behavioural alterations in mice: implications for experimental psychosis. Behav. Brain Res. 216, 247–254. 10.1016/j.bbr.2010.08.001 20699106

[B24] Choy K. H. C. Dean O. Berk M. Bush A. I. van den Buuse M. (2010). Effects of *N*-acetyl-cysteine treatment on glutathione depletion and a short-term spatial memory deficit in 2-cyclohexene-1-one-treated rats. Eur. J. Pharmacol. 649, 224–228. 10.1016/j.ejphar.2010.09.035 20868666

[B25] Coleman L. G. Jr. Jarskog L. F. Moy S. S. Crews F. T. (2009). Deficits in adult prefrontal cortex neurons and behavior following early post-natal NMDA antagonist treatment. Pharmacol. Biochem. Behav. 93, 322–330. 10.1016/j.pbb.2009.04.017 19409920 PMC2798004

[B26] de Lima M. N. M. Laranja D. C. Bromberg E. Roesler R. Schröder N. (2005). Pre- or post-training administration of the NMDA receptor blocker MK-801 impairs object recognition memory in rats. Behav. Brain Res. 156, 139–143. 10.1016/j.bbr.2004.05.016 15474658

[B27] Do K. Q. Cabungcal J. H. Frank A. Steullet P. Cuenod M. (2009). Redox dysregulation, neurodevelopment and schizophrenia. Curr. Opin. Neurobiol. 19, 220–230. 10.1016/j.conb.2009.05.001 19481443

[B28] Do K. Q. Cuenod M. Hensch T. K. (2015). Targeting oxidative stress and aberrant critical period plasticity in the developmental trajectory to schizophrenia. Schizophr. Bull. 41, 835–846. 10.1093/schbul/sbv065 26032508 PMC4466197

[B29] Do K. Q. Trabesinger A. H. Kirsten-Krüger M. Lauer C. J. Dydak U. Hell D.. (2000). Schizophrenia: glutathione deficit in cerebrospinal fluid and prefrontal cortex *in vivo*. Eur. J. Neurosci. 12, 3721–3728. 10.1046/j.1460-9568.2000.00229.x 11029642

[B30] du Bois T. M. Huang X. F. (2007). Early brain development disruption from NMDA receptor hypofunction: relevance to schizophrenia. Brain Res. Rev. 53, 260–270. 10.1016/j.brainresrev.2006.09.001 17014910

[B31] Eling P. Derckx K. Maes R. (2008). On the historical and conceptual background of the Wisconsin Card Sorting Test. Brain Cogn. 67, 247–253. 10.1016/j.bandc.2008.01.006 18328609

[B32] Ellinwood E. H. Jr. Balster R. L. (1974). Rating the behavioral effects of amphetamine. Eur. J. Pharmacol. 28, 35–41. 10.1016/0014-2999(74)90109-5 4473346

[B33] Farokhnia M. Azarkolah A. Adinehfar F. Khodaie-Ardakani M. R. Hosseini S. M. Yekehtaz H.. (2013). *N*-acetylcysteine as an adjunct to risperidone for treatment of negative symptoms in patients with chronic schizophrenia: a randomized, double-blind, placebo-controlled study. Clin. Neuropharmacol. 36, 185–192. 10.1097/WNF.0000000000000001 24201233

[B35] Flatow J. Buckley P. Miller B. J. (2013). Meta-analysis of oxidative stress in schizophrenia. Biol. Psychiatry 74, 400–409. 10.1016/j.biopsych.2013.03.018 23683390 PMC4018767

[B36] Floresco S. B. Magyar O. Ghods-Sharifi S. Vexelman C. Tse M. T. (2006). Multiple dopamine receptor subtypes in the medial prefrontal cortex of the rat regulate set-shifting. Neuropsychopharmacology 31, 297–309. 10.1038/sj.npp.1300825 16012531

[B37] Floresco S. B. Zhang Y. Enomoto T. (2009). Neural circuits subserving behavioral flexibility and their relevance to schizophrenia. Behav. Brain Res. 204, 396–409. 10.1016/j.bbr.2008.12.001 19110006

[B38] Gawryluk J. W. Wang J.-F. Andreazza A. C. Shao L. Young L. T. (2011). Decreased levels of glutathione, the major brain antioxidant, in post-mortem prefrontal cortex from patients with psychiatric disorders. Int. J. Neuropsychopharmacol. 14, 123–130. 10.1017/S1461145710000805 20633320

[B39] Geyer M. A. Markou A. (1995). “Animal models of psychiatric disorders,” in Psychopharmacology—The Fourth Generation of Progress, eds Bloom F. E. Kupfer D. J. (New York, NY: Raven Press), 787–798.

[B40] Grayson B. Barnes S. A. Markou A. Piercy C. Podda G. Neill J. C. (2016). Postnatal phencyclidine (PCP) as a neurodevelopmental animal model of schizophrenia pathophysiology and symptomatology: a review. Curr. Top. Behav. Neurosci. 29, 403–428. 10.1007/7854_2015_403 26510740

[B41] Grayson B. Idris N. F. Neill J. C. (2007). Atypical antipsychotics attenuate a sub-chronic PCP-induced cognitive deficit in the novel object recognition task in the rat. Behav. Brain Res. 184, 31–38. 10.1016/j.bbr.2007.06.012 17675172

[B42] Green M. (1996). What are the functional consequences of neurocognitive deficits in schizophrenia? Am. J. Psychiatry 153, 321–330. 10.1176/ajp.153.3.321 8610818

[B43] Green M. F. Kern R. S. Heaton R. K. (2004). Longitudinal studies of cognition and functional outcome in schizophrenia: implications for MATRICS. Schizophr. Res. 72, 41–51. 10.1016/j.schres.2004.09.009 15531406

[B45] Gysin R. Kraftsik R. Sandell J. Bovet P. Chappuis C. Conus P.. (2007). Impaired glutathione synthesis in schizophrenia: convergent genetic and functional evidence. Proc. Natl. Acad. Sci. U S A 104, 16621–16666. 10.1073/pnas.0706778104 17921251 PMC2034265

[B46] Häfner H. Löffler W. Maurer K. Hambrecht M. an der Heiden W. (1999). Depression, negative symptoms, social stagnation and social decline in the early course of schizophrenia. Acta Psych. Scand. 100, 105–118. 10.1111/j.1600-0447.1999.tb10831.x 10480196

[B47] Hardingham G. E. Do K. Q. (2016). Linking early-life NMDAR hypofunction and oxidative stress in schizophrenia pathogenesis. Nat. Rev. Neurosci. 17, 125–134. 10.1038/nrn.2015.19 26763624

[B48] Harich S. Gross G. Bespalov A. (2007). Stimulation of the metabotropic glutamate 2/3 receptor attenuates social novelty discrimination deficits induced by neonatal phencyclidine treatment. Psychopharmacology 192, 511–519. 10.1007/s00213-007-0742-y 17318501

[B49] Harvey B. H. Joubert C. du Preez J. L. Berk M. (2008). Effect of chronic N-acetyl cysteine administration on oxidative status in the presence and absence of induced oxidative stress in rat striatum. Neurochem. Res. 33, 508–517. 10.1007/s11064-007-9466-y 17763945

[B50] Hashimoto K. Fujita Y. Iyo M. (2007). Phencyclidine-induced cognitive deficits in mice are improved by subsequent subchronic administration of fluvoxamine: role of sigma-1 receptors. Neuropsychopharmacology 32, 514–521. 10.1038/sj.npp.1301047 16495935

[B51] Heckers S. Curran T. Goff D. Rauch S. L. Fischman A. J. Alpert N. M.. (2000). Abnormalities in the thalamus and prefrontal cortex during episodic object recognition in schizophrenia. Biol. Psychiatry 48, 651–657. 10.1016/s0006-3223(00)00919-7 11032976

[B52] Hill R. A. (2016). Sex differences in animal models of schizophrenia shed light on the underlying pathophysiology. Neurosci. Biobehav. Rev. 67, 41–56. 10.1016/j.neubiorev.2015.10.014 26743857

[B53] Howes O. D. Kapur S. (2009). The dopamine hypothesis of schizophrenia: version III—the final common pathway. Schizophr. Bull. 35, 549–562. 10.1093/schbul/sbp006 19325164 PMC2669582

[B54] Javitt D. C. Zukin S. R. (1991). Recent advances in the phencyclidine model of schizophrenia. Am. J. Psychiatry 148, 1301–1308. 10.1176/ajp.148.10.1301 1654746

[B56] Jeevakumar V. Driskill C. Paine A. Sobhanian M. Vakil H. Morris B.. (2015). Ketamine administration during the second postnatal week induces enduring schizophrenia-like behavioral symptoms and reduces parvalbumin expression in the medial prefrontal cortex of adult mice. Behav. Brain Res. 282, 165–175. 10.1016/j.bbr.2015.01.010 25591475

[B55] Jeevakumar V. Kroener S. (2016). Ketamine administration during the second postnatal week alters synaptic properties of fast-spiking interneurons in the medial prefrontal cortex of adult mice. Cereb. Cortex 26, 1117–1129. 10.1093/cercor/bhu293 25477370

[B57] Johnstone E. C. Ebmeier K. P. Miller P. Owens D. G. C. Lawrie S. M. (2005). Predicting schizophrenia: findings from the Edinburgh High-Risk study. Br. J. Psychiatry 186, 18–25. 10.1192/bjp.186.1.18 15630119

[B58] Jones K. S. Corbin J. G. Huntsman M. M. (2014). Neonatal NMDA receptor blockade disrupts spike timing and glutamatergic synapses in fast spiking interneurons in a NMDA receptor hypofunction model of schizophrenia. PLoS One 9:e109303. 10.1371/journal.pone.0109303 25290690 PMC4188593

[B59] Kahn R. S. Keefe R. S. E. (2013). Schizophrenia is a cognitive illness. JAMA Psychiatry 70, 1107–1112. 10.1001/jamapsychiatry.2013.155 23925787

[B60] Kantrowitz J. T. Epstein M. L. Beggel O. Rohrig S. Lehrfeld J. M. Revheim N.. (2016). Neurophysiological mechanisms of cortical plasticity impairments in schizophrenia and modulation by the NMDA receptor agonist D-serine. Brain 139, 3281–3295. 10.1093/brain/aww262 27913408 PMC5840885

[B61] Karper L. P. Freeman G. K. Grillon C. Morgan C. A. III Charney D. S. Krystal J. H. (1996). Preliminary evidence of an association between sensorimotor gating and distractibility in psychosis. J. Neuropsychiatry Clin. Neurosci. 8, 60–66. 10.1176/jnp.8.1.60 8845703

[B62] Keefe R. S. E. Harvey P. D. (2012). “Cognitive impairment in schizophrenia,” in Novel Antischizophrenia Treatments, eds Geyer M. A. Gross G. (Berlin, Heidelberg: Springer), 11–37.

[B63] Kröller-Schön S. Steven S. Kossmann S. Scholz A. Daub S. Oelze M.. (2014). Molecular mechanisms of the crosstalk between mitochondria and NADPH oxidase through reactive oxygen species—studies in white blood cells and in animal models. Antioxid. Redox Signal. 20, 247–266. 10.1089/ars.2012.4953 23845067 PMC3887465

[B64] Krystal J. H. Anand A. Moghaddam B. (2002). Effects of NMDA receptor antagonists: implications for the pathophysiology of schizophrenia. Arch. Gen. Psychiatry 59, 663–664. 10.1001/archpsyc.59.7.663 12090822

[B65] Lahti A. C. Koffel B. LaPorte D. Tamminga C. A. (1995). Subanesthetic doses of ketamine stimulate psychosis in schizophrenia. Neuropsychopharmacology 13, 9–19. 10.1038/sj.npp.1380271 8526975

[B66] Lalonde R. (2002). The neurobiological basis of spontaneous alternation. Neurosci. Biobehav. Rev. 26, 91–104. 10.1016/s0149-7634(01)00041-0 11835987

[B67] Laruelle M. Abi-Dargham A. van Dyck C. H. Gil R. D’Souza C. D. Erdos J.. (1996). Single photon emission computerized tomography imaging of amphetamine-induced dopamine release in drug-free schizophrenic subjects. Proc. Natl. Acad. Sci. U S A 93, 9235–9240. 10.1073/pnas.93.17.9235 8799184 PMC38625

[B68] Lavoie S. Murray M. M. Deppen P. Knyazeva M. G. Berk M. Boulat O.. (2008). Glutathione precursor, *N*-acetyl-cysteine, improves mismatch negativity in schizophrenia patients. Neuropsychopharmacology 33, 2187–2199. 10.1038/sj.npp.1301624 18004285

[B69] Law A. J. Deakin J. F. (2001). Asymmetrical reductions of hippocampal NMDAR1 glutamate receptor mRNA in the psychoses. Neuroreport 12, 2971–2974. 10.1097/00001756-200109170-00043 11588613

[B70] Leger M. Neill J. C. (2016). A systematic review comparing sex differences in cognitive function in schizophrenia and in rodent models for schizophrenia, implications for improved therapeutic strategies. Neurosci. Biobehav. Rev. 68, 979–1000. 10.1016/j.neubiorev.2016.06.029 27344000

[B71] Lewis D. A. Curley A. A. Glausier J. R. Volk D. W. (2012). Cortical parvalbumin interneurons and cognitive dysfunction in schizophrenia. Trends Neurosci. 35, 57–67. 10.1016/j.tins.2011.10.004 22154068 PMC3253230

[B72] Lipska B. K. Swerdlow N. R. Geyer M. A. Jaskiw G. E. Braff D. L. Weinberger D. R. (1995). Neonatal excitotoxic hippocampal damage in rats causes post-pubertal changes in prepulse inhibition of startle and its disruption by apomorphine. Psychopharmacology 122, 35–43. 10.1007/bf02246439 8711062

[B73] Lutgen V. Qualmann K. Resch J. Kong L. Choi S. Baker D. A. (2013). Reduction in phencyclidine induced sensorimotor gating deficits in the rat following increased system xc^−^ activity in the medial prefrontal cortex. Psychopharmacology 226, 531–540. 10.1007/s00213-012-2926-3 23192314 PMC3595356

[B74] Lyall A. Swanson J. Liu C. Blumenthal T. D. Turner C. P. (2009). Neonatal exposure to MK801 promotes prepulse-induced delay in startle response time in adult rats. Exp. Brain Res. 197, 215–222. 10.1007/s00221-009-1906-2 19565228 PMC2752751

[B75] Mahadik S. P. Mukherjee S. (1996). Free radical pathology and antioxidant defense in schizophrenia: a review. Schizophr. Res. 19, 1–17. 10.1016/0920-9964(95)00049-6 9147491

[B76] Mailman R. B. Murthy V. (2010). Third generation antipsychotic drugs: partial agonism or receptor functional selectivity? Curr. Pharm. Des. 16, 488–501. 10.2174/138161210790361461 19909227 PMC2958217

[B77] Malhotra A. K. Pinals D. A. Adler C. M. Elman I. Clifton A. Pickar D.. (1997). Ketamine-induced exacerbation of psychotic symptoms and cognitive impairment in neuroleptic-free schizophrenics. Neuropsychopharmacology 17, 141–150. 10.1016/s0893-133x(97)00036-5 9272481

[B78] Marder S. R. (2006). The NIMH-MATRICS project for developing cognition-enhancing agents for schizophrenia. Dialogues Clin. Neurosci. 8, 109–113. 16640121 10.31887/DCNS.2006.8.1/smarder PMC3181758

[B79] Massaad C. A. Klann E. (2011). Reactive oxygen species in the regulation of synaptic plasticity and memory. Antioxid. Redox Signal. 14, 2013–2054. 10.1089/ars.2010.3208 20649473 PMC3078504

[B80] Matsuzawa D. Hashimoto K. (2011). Magnetic resonance spectroscopy study of the antioxidant defense system in schizophrenia. Antioxid. Redox Signal. 15, 2057–2065. 10.1089/ars.2010.3453 20712400

[B81] McClellan J. Breiger D. McCurry C. Hlastala S. (2003). Premorbid functioning in early-onset psychotic disorders. J. Am. Acad. Child Adolesc. Psychiatry 42, 666–672. 10.1097/01.chi.0000046844.56865.6b 12921474

[B83] Møller P. Husby R. (2000). The initial prodrome in schizophrenia: searching for naturalistic core dimensions of experience and behavior. Schizophr. Bull. 26, 217–232. 10.1093/oxfordjournals.schbul.a033442 10755683

[B82] Möller M. Swanepoel T. Harvey B. H. (2015). Neurodevelopmental animal models reveal the convergent role of neurotransmitter systems, inflammation and oxidative stress as biomarkers of schizophrenia: implications for novel drug development. ACS Chem. Neurosci. 6, 987–1016. 10.1021/cn5003368 25794269

[B84] Morici J. F. Bekinschtein P. Weisstaub N. V. (2015). Medial prefrontal cortex role in recognition memory in rodents. Behav. Brain Res. 292, 241–251. 10.1016/j.bbr.2015.06.030 26115848

[B85] Moy S. S. Nadler J. J. Perez A. Barbaro R. P. Johns J. M. Magnuson T. R.. (2004). Sociability and preference for social novelty in five inbred strains: an approach to assess autistic-like behavior in mice. Genes Brain Behav. 3, 287–302. 10.1111/j.1601-1848.2004.00076.x 15344922

[B86] Nagai T. Murai R. Matsui K. Kamei H. Noda Y. Furukawa H.. (2009). Aripiprazole ameliorates phencyclidine-induced impairment of recognition memory through dopamine D1 and serotonin 5-HT1A receptors. Psychopharmacology 202, 315–328. 10.1007/s00213-008-1240-6 18679658

[B87] Neill J. C. Barnes S. Cook S. Grayson B. Idris N. F. McLean S. L.. (2010). Animal models of cognitive dysfunction and negative symptoms of schizophrenia: focus on NMDA receptor antagonism. Pharmacol. Ther. 128, 419–432. 10.1016/j.pharmthera.2010.07.004 20705091

[B88] Nieuwenstein M. R. Aleman A. de Haan E. H. F. (2001). Relationship between symptom dimensions and neurocognitive functioning in schizophrenia: a meta-analysis of WCST and CPT studies. J. Psychiatr. Res. 35, 119–125. 10.1016/s0022-3956(01)00014-0 11377441

[B89] Nikiforuk A. Gołembiowska K. Popik P. (2010). Mazindol attenuates ketamine-induced cognitive deficit in the attentional set shifting task in rats. Eur. Neuropsychopharmacol. 20, 37–48. 10.1016/j.euroneuro.2009.08.001 19729284

[B90] Nikiforuk A. Kos T. Fijał K. Hołuj M. Rafa D. Popik P. (2013). Effects of the selective 5-HT7 receptor antagonist SB-269970 and amisulpride on ketamine-induced schizophrenia-like deficits in rats. PLoS One 8:e66695. 10.1371/journal.pone.0066695 23776692 PMC3679060

[B91] Nuechterlein K. H. Barch D. M. Gold J. M. Goldberg T. E. Green M. F. Heaton R. K. (2004). Identification of separable cognitive factors in schizophrenia. Schizophr. Res. 72, 29–39. 10.1016/j.schres.2004.09.007 15531405

[B92] Pantelis C. Barber F. Z. Barnes T. R. Nelson H. E. Owen A. M. Robbins T. W. (1999). Comparison of set-shifting ability in patients with chronic schizophrenia and frontal lobe damage. Schizophr. Res. 37, 251–270. 10.1016/s0920-9964(98)00156-x 10403197

[B93] Perry W. Braff D. L. (1994). Information-processing deficits and thought disorder in schizophrenia. Am. J. Psychiatry 151, 363–367. 10.1176/ajp.151.3.363 8109644

[B94] Perry W. Geyer M. A. Braff D. L. (1999). Sensorimotor gating and thought disturbance measured in close temporal proximity in schizophrenic patients. Arch. Gen. Psychiatry 56, 277–281. 10.1001/archpsyc.56.3.277 10078506

[B95] Pitsikas N. Boultadakis A. Sakellaridis N. (2008). Effects of sub-anesthetic doses of ketamine on rats’ spatial and non-spatial recognition memory. Neuroscience 154, 454–460. 10.1016/j.neuroscience.2008.04.001 18472348

[B96] Powell S. B. (2010). Models of neurodevelopmental abnormalities in schizophrenia. Curr. Top. Behav. Neurosci. 4, 435–481. 10.1007/7854_2010_57 21312409 PMC3595002

[B97] Powell S. B. Sejnowski T. J. Behrens M. M. (2012a). Behavioral and neurochemical consequences of cortical oxidative stress on parvalbumin-interneuron maturation in rodent models of schizophrenia. Neuropharmacology 62, 1322–1331. 10.1016/j.neuropharm.2011.01.049 21315745 PMC3106123

[B98] Powell S. B. Weber M. Geyer M. A. (2012b). Genetic models of sensorimotor gating: relevance to neuropsychiatric disorders. Curr. Top. Behav. Neurosci. 12, 251–318. 10.1007/7854_2011_195 22367921 PMC3357439

[B99] Prabakaran S. Swatton J. E. Ryan M. M. Huffaker S. J. Huang J. T.-J. Griffin J. L.. (2004). Mitochondrial dysfunction in schizophrenia: evidence for compromised brain metabolism and oxidative stress. Mol. Psychiatry 9, 684–697. 10.1038/sj.mp.4001511 15098003

[B100] Ragozzino M. E. (2002). The effects of dopamine D_1_ receptor blockade in the prelimbic-infralimbic areas on behavioral flexibility. Learn. Mem. 9, 18–28. 10.1101/lm.45802 11917003 PMC155930

[B101] Rajagopal L. Massey B. Huang M. Oyamada Y. Meltzer H. (2014). The novel object recognition test in rodents in relation to cognitive impairment in schizophrenia. Curr. Pharm. Des. 20, 5104–5114. 10.2174/1381612819666131216114240 24345269

[B102] Rajasekaran A. Venkatasubramanian G. Berk M. Debnath M. (2015). Mitochondrial dysfunction in schizophrenia: pathways, mechanisms and implications. Neurosci. Biobehav. Rev. 48, 10–21. 10.1016/j.neubiorev.2014.11.005 25446950

[B103] Rapado-Castro M. Dodd S. Bush A. I. Malhi G. S. Skvarc D. R. On Z. X.. (2017). Cognitive effects of adjunctive *N*-acetyl cysteine in psychosis. Psych. Med. 47, 866–876. 10.1017/s0033291716002932 27894373

[B104] Robinson T. E. Becker J. B. (1986). Enduring changes in brain and behavior produced by chronic amphetamine administration: a review and evaluation of animal models of amphetamine psychosis. Brain Res. 396, 157–198. 10.1016/s0006-8993(86)80193-7 3527341

[B105] Sams-Dodd F. (1998). Effects of dopamine agonists and antagonists on PCP-induced stereotyped behaviour and social isolation in the rat social interaction test. Psychopharmacology 135, 182–193. 10.1007/s002130050500 9497024

[B106] Schiavone S. Sorce S. Dubois-Dauphin M. Jaquet V. Colaianna M. Zotti M.. (2009). Involvement of NOX2 in the development of behavioral and pathologic alterations in isolated rats. Biol. Psychiatry 66, 384–392. 10.1016/j.biopsych.2009.04.033 19559404

[B107] Segal D. S. Geyer M. A. Schuckit M. A. (1981). Stimulant-induced psychosis: an evaluation of animal methods. Essays Neurochem. Neuropharmacol. 5, 95–129. 10.1002/9781444314571.ch11 6112147

[B108] Sorce S. Schiavone S. Tucci P. Colaianna M. Jaquet V. Cuomo V.. (2010). The NADPH oxidase NOX2 controls glutamate release: a novel mechanism involved in psychosis-like ketamine responses. J. Neurosci. 30, 11317–11325. 10.1523/JNEUROSCI.1491-10.2010 20739552 PMC6633347

[B110] Stefani M. R. Groth K. Moghaddam B. (2003). Glutamate receptors in the rat medial prefrontal cortex regulate set-shifting ability. Behav. Neurosci. 117, 728–737. 10.1037/0735-7044.117.4.728 12931958

[B109] Stefani M. R. Moghaddam B. (2005). Transient N-methyl-D-aspartate receptor blockade in early development causes lasting cognitive deficits relevant to schizophrenia. Biol. Psychiatry 57, 433–436. 10.1016/j.biopsych.2004.11.031 15705361

[B111] Steullet P. Cabungcal J.-H. Kulak A. Kraftsik R. Chen Y. Dalton T. P.. (2010). Redox dysregulation affects the ventral but not dorsal hippocampus: impairment of parvalbumin neurons, γ oscillations, and related behaviors. J. Neurosci. 30, 2547–2558. 10.1523/JNEUROSCI.3857-09.2010 20164340 PMC6634545

[B112] Stone W. S. Hsi X. (2011). Declarative memory deficits and schizophrenia: problems and prospects. Neurobiol. Learn. Mem. 96, 544–552. 10.1016/j.nlm.2011.04.006 21527348

[B200] Swerdlow N. R. Geyer M. A. Braff D. L. (2001). Neural circuit regulation of prepulse inhibition of startle in the rat: current knowledge and future challenges. Psychopharmacology 156, 194–215. 10.1007/s002130100799 11549223

[B113] Swerdlow N. R. Vaccarino F. J. Amalric M. Koob G. F. (1986). The neural substrates for the motor-activating properties of psychostimulants: a review of recent findings. Pharmacol. Biochem. Behav. 25, 233–248. 10.1016/0091-3057(86)90261-3 2875470

[B114] Terranova J.-P. Chabot C. Barnouin M.-C. Perrault G. Depoortere R. Griebel G.. (2005). SSR181507, a dopamine D_2_ receptor antagonist and 5-HT_1A_ receptor agonist, alleviates disturbances of novelty discrimination in a social context in rats, a putative model of selective attention deficit. Psychopharmacology 181, 134–144. 10.1007/s00213-005-2268-5 15830220

[B115] Tosic M. Ott J. Barral S. Bovet P. Deppen P. Gheorghita F.. (2006). Schizophrenia and oxidative stress: glutamate cysteine ligase modifier as a susceptibility gene. Am. J. Human Genet. 79, 586–592. 10.1086/507566 16909399 PMC1559555

[B116] van den Buuse M. (2010). Modeling the positive symptoms of schizophrenia in genetically modified mice: pharmacology and methodology aspects. Schizophr. Bull. 36, 246–270. 10.1093/schbul/sbp132 19900963 PMC2833124

[B117] Venâncio C. Magalhães A. Antunes L. Summavielle T. (2011). Impaired spatial memory after ketamine administration in chronic low doses. Curr. Neuropharmacol. 9, 251–255. 10.2174/157015911795016912 21886600 PMC3137193

[B118] Wang C. McInnis J. West J. B. Bao J. Anastasio N. Guidry J. A.. (2003). Blockade of phencyclidine-induced cortical apoptosis and deficits in prepulse inhibition by M40403, a superoxide dismutase mimetic. J. Pharmacol. Exp. Ther. 304, 266–271. 10.1124/jpet.102.041798 12490600

[B119] Wang D. Noda Y. Zhou Y. Nitta A. Furukawa H. Nabeshima T. (2007). Synergistic effect of galantamine with risperidone on impairment of social interaction in phencyclidine-treated mice as a schizophrenic animal model. Neuropharmacology 52, 1179–1187. 10.1016/j.neuropharm.2006.12.007 17313962

[B120] Wedzony K. Fijał K. Mackowiak M. Chocyk A. (2008). Detrimental effect of postnatal blockade of *N*-methyl-D-aspartate receptors on sensorimotor gating is reversed by neuroleptic drugs. Pharmacol. Rep. 60, 856–864. 19211977

[B121] Weiss A. Heckers S. (2001). Neuroimaging of declarative memory in schizophrenia. Scand. J. Psychol. 42, 239–250. 10.1111/1467-9450.00234 11501738

[B122] Wenzel P. Mollnau H. Oelze M. Schulz E. Wickramanayake J. M. D. Müller J.. (2008). First evidence for a crosstalk between mitochondrial and NADPH oxidase-derived reactive oxygen species in nitroglycerin-triggered vascular dysfunction. Antioxid. Redox Signal. 10, 1435–1448. 10.1089/ars.2007.1969 18522491

[B123] Xia S. Cai Z. Y. Thio L. L. Kim-Han J. S. Dugan L. L. Covey D. F.. (2002). The estrogen receptor is not essential for all estrogen neuroprotection: new evidence from a new analog. Neurobiol. Dis. 9, 282–293. 10.1006/nbdi.2002.0478 11950274

[B124] Yao J. K. Leonard S. Reddy R. (2006). Altered glutathione redox state in schizophrenia. Dis. Markers 22, 83–93. 10.1155/2006/248387 16410648 PMC3850558

[B126] Young J. W. Amitai N. Geyer M. A. (2012). Behavioral animal models to assess pro-cognitive treatments for schizophrenia. Handb. Exp. Pharmacol. 213, 39–79. 10.1007/978-3-642-25758-2_3 23027412

[B125] Young J. W. Powell S. B. Geyer M. A. Jeste D. V. Risbrough V. B. (2010). The mouse attentional-set-shifting task: a method for assaying successful cognitive aging? Cogn. Affect. Behav. Neurosci. 10, 243–251. 10.3758/CABN.10.2.243 20498348 PMC2877277

[B127] Young J. W. Powell S. B. Risbrough V. Marston H. M. Geyer M. A. (2009). Using the MATRICS to guide development of a preclinical cognitive test battery for research in schizophrenia. Pharmacol. Ther. 122, 150–202. 10.1016/j.pharmthera.2009.02.004 19269307 PMC2688712

[B128] Zhang X. Y. Chen D. C. Xiu M. H. Tang W. Zhang F. Liu L.-J.. (2012). Plasma total antioxidant status and cognitive impairments in schizophrenia. Schizophr. Res. 139, 66–72. 10.1016/j.schres.2012.04.009 22555016

[B129] Zhang X. Y. Chen D. C. Xiu M. H. Yang F. D. Tan Y. L. He S.. (2013). Thioredoxin, a novel oxidative stress marker and cognitive performance in chronic and medicated schizophrenia versus healthy controls. Schizophr. Res. 143, 301–306. 10.1016/j.schres.2012.11.017 23238053

[B130] Zuo D.-Y. Wu Y.-L. Yao W.-X. Cao Y. Wu C.-F. Tanaka M. (2007). Effect of MK-801 and ketamine on hydroxyl radical generation in the posterior cingulate and retrosplenial cortex of free-moving mice, as determined by *in vivo* microdialysis. Pharmacol. Biochem. Behav. 86, 1–7. 10.1016/j.pbb.2006.05.010 16806445

